# EEG–motor correlation as early Alzheimer’s disease index in herpes simplex virus type-1–infected mice

**DOI:** 10.1093/braincomms/fcag128

**Published:** 2026-04-10

**Authors:** Chiara D’Amelio, Chiara Feroleto, Chiara Caligiuri, Domenica Donatella Li Puma, Giovanna De Chiara, Ilaria Paoletti, Camilla Codazzi, Federica D’Alelio, Francesca Miraglia, Chiara Pappalettera, Lorenzo Nucci, Federico Frasca, Lucia Ventura, Andrea Manca, Marco Morrone, Lucia Leone, Franca Deriu, Marta Morotti, Claudio Grassi, Fabrizio Vecchio, Maria Vittoria Podda

**Affiliations:** Department of Neuroscience, Università Cattolica del Sacro Cuore, Rome 00168, Italy; Department of Neuroscience, Università Cattolica del Sacro Cuore, Rome 00168, Italy; Fondazione Policlinico Universitario A. Gemelli IRCCS, Rome 00168, Italy; Department of Neuroscience, Università Cattolica del Sacro Cuore, Rome 00168, Italy; Department of Neuroscience, Università Cattolica del Sacro Cuore, Rome 00168, Italy; Fondazione Policlinico Universitario A. Gemelli IRCCS, Rome 00168, Italy; Institute of Translational Pharmacology, Natl. Res. Council, Rome 00133, Italy; Fondazione Policlinico Universitario A. Gemelli IRCCS, Rome 00168, Italy; Department of Neuroscience, Università Cattolica del Sacro Cuore, Rome 00168, Italy; Fondazione Policlinico Universitario A. Gemelli IRCCS, Rome 00168, Italy; Brain Connectivity Laboratory, Department of Neuroscience and Neurorehabilitation, IRCCS San Raffaele, Rome 00166, Italy; Department of Theoretical and Applied Sciences, eCampus University, Novedrate, Como 22060, Italy; Brain Connectivity Laboratory, Department of Neuroscience and Neurorehabilitation, IRCCS San Raffaele, Rome 00166, Italy; Brain Connectivity Laboratory, Department of Neuroscience and Neurorehabilitation, IRCCS San Raffaele, Rome 00166, Italy; Brain Connectivity Laboratory, Department of Neuroscience and Neurorehabilitation, IRCCS San Raffaele, Rome 00166, Italy; Azienda Ospedaliero Universitaria di Sassari, Sassari 07100, Italy; Department of Biomedical Sciences, University of Sassari, Sassari 07100, Italy; Department of Biomedical Sciences, University of Sassari, Sassari 07100, Italy; Department of Biomedical Sciences, University of Sassari, Sassari 07100, Italy; Department of Neuroscience, Università Cattolica del Sacro Cuore, Rome 00168, Italy; Fondazione Policlinico Universitario A. Gemelli IRCCS, Rome 00168, Italy; Azienda Ospedaliero Universitaria di Sassari, Sassari 07100, Italy; Department of Biomedical Sciences, University of Sassari, Sassari 07100, Italy; Unit of Endocrinology, Nutrition, and Metabolic Disorders, AOUSS, Sassari 07100, Italy; Fondazione Policlinico Universitario A. Gemelli IRCCS, Rome 00168, Italy; Department of Neuroscience, Università Cattolica del Sacro Cuore, Rome 00168, Italy; Fondazione Policlinico Universitario A. Gemelli IRCCS, Rome 00168, Italy; Brain Connectivity Laboratory, Department of Neuroscience and Neurorehabilitation, IRCCS San Raffaele, Rome 00166, Italy; Department of Theoretical and Applied Sciences, eCampus University, Novedrate, Como 22060, Italy; Department of Neuroscience, Università Cattolica del Sacro Cuore, Rome 00168, Italy; Fondazione Policlinico Universitario A. Gemelli IRCCS, Rome 00168, Italy

**Keywords:** Alzheimer’s disease, EEG biomarker, functional connectivity, mild cognitive impairment, motor dysfunction

## Abstract

Alzheimer's disease is a neurodegenerative disorder characterized by cognitive decline and memory impairment. Early treatment requires reliable tests to identify the initial manifestations for developing treatments that modify disease progression. Neuroinflammation has been implicated as a key driver of the onset and progression of Alzheimer's disease. Herpes simplex virus type-1 (HSV-1), a neurotropic virus that establishes latency within the central nervous system, has been associated with increased proinflammatory cytokines, cognitive impairment and Alzheimer's disease–like pathology in human and rodent brains. This study employed a murine model showing an Alzheimer's disease–related phenotype, induced by HSV-1 infection and recurrent reactivation through thermal stress, to investigate previously unexplored motor function impairments and their correlation with EEG changes predictive of Alzheimer's disease–like pathology. Mice were subjected to two (2×TS) or seven thermal stress (7×TS) HSV-1 reactivations to reproduce mild and severe cognitive impairments, respectively, and were tested for recognition memory using the Novel Object Recognition test and for spatial memory using the Y-maze test. Motor performance was assessed using grip strength and grid walking tests. Local field potential recordings, immunohistochemical, morphological and molecular analyses were performed to characterize the effects of HSV-1 on neural circuits. 2×TS HSV-1 mice showed a reduced preference index in Novel Object Recognition compared to mice receiving mock infection (i.e. vehicle inoculum), whereas 7×TS HSV-1 mice displayed severe cognitive decline across the different memory domains. Motor function was preserved after the second thermal stress but was impaired after the seventh thermal stress, with reduced forelimb force and increased foot faults starting from the fourth reactivation. Following the seventh reactivation, HSV-1 mice showed astrogliosis and phosphorylated Tau accumulation. *In vivo*, electrophysiological recordings revealed increased functional connectivity across frequency bands in 2×TS HSV-1 mice compared to controls, with negative correlations between total coherence and grip strength. Increased spine density in the frontal cortex of 2×TS HSV-1 mice supports early neuronal network alterations. From a translational perspective, we preliminarily evaluated comparable motor indices in healthy human participants, in patients with mild cognitive impairment, and in patients with Alzheimer's disease. As expected, both grip strength and dynamic balance were lower in patients with Alzheimer's disease compared to healthy and mild cognitive impairment subjects. Notably, grip strength was significantly reduced in mild cognitive impairment subjects, who displayed early motor impairment. Our findings highlight the potential of EEG-based biomarkers for early Alzheimer's disease detection and suggest motor indices as novel prognostic markers.

## Teaser

D’Amelio et al. used a mouse model in which herpes simplex virus type-1 reactivations drive the development of Alzheimer’s disease–like phenotype. Mice with mild cognitive impairment showed network hyperconnectivity correlating with motor deficits. Similar motor dysfunctions appeared in mild cognitive impairment patients, supporting motor and EEG indices as prognostic markers.

## Graphical Abstract

**Graphical Abstract:**
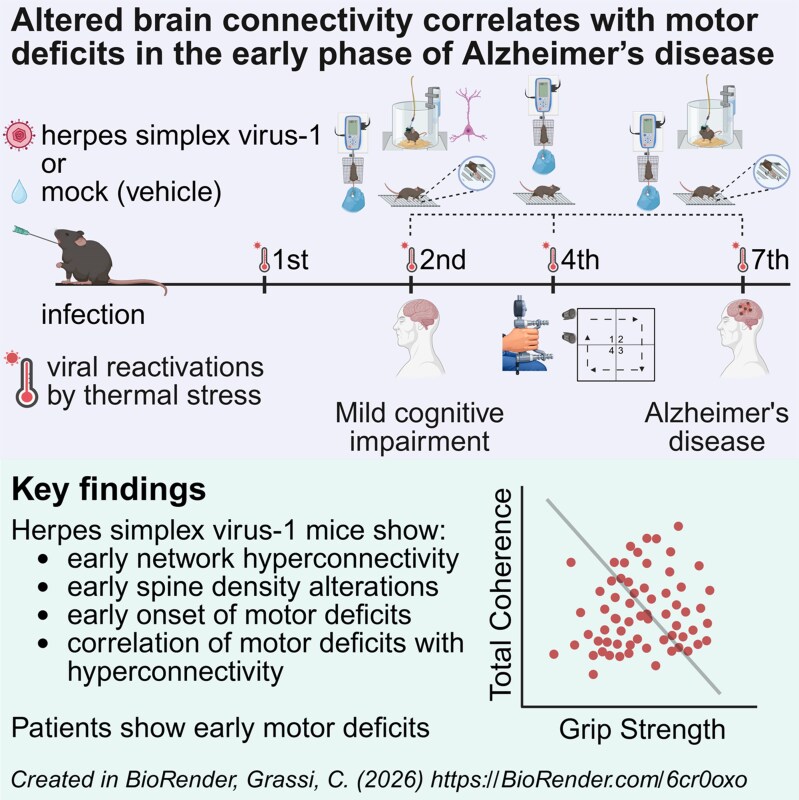

## Introduction

Alzheimer's disease is a multifactorial neurodegenerative disorder in which the non-linear interaction between genetic, biological and environmental factors accounts for its inherent inter-individual clinical variability.^1^ Alzheimer's disease is characterized by cognitive decline, memory impairment and behavioural changes. The hallmarks of Alzheimer's disease include extracellular amyloid-beta (Aβ) plaques, intracellular neurofibrillary tangles composed of hyperphosphorylated Tau and widespread neuroinflammation.^2^ Although the aetiology of sporadic Alzheimer's disease remains multifactorial and incompletely understood, accumulating evidence implicates inflammation as a key driver of disease onset and progression.^3^

Among the potential contributors to neuroinflammatory processes in Alzheimer's disease, herpes simplex virus type-1 (HSV-1) has attracted significant attention. HSV-1 is a neurotropic virus capable of establishing latency within the central nervous system, particularly in the trigeminal ganglia and temporal lobes, which are notably vulnerable to Alzheimer's disease.^4,5^ Repeated reactivation of latent HSV-1 has been associated with increased expression of proinflammatory cytokines, microglial and astrocyte activation and exacerbation of Alzheimer's disease–like pathology in both human and animal studies.^6-10^ These findings support the hypothesis that recurrent viral infections act as environmental triggers for Alzheimer's disease, potentially accelerating neurodegeneration in genetically or environmentally susceptible individuals.

Despite advances in our understanding of the molecular and cellular mechanisms underlying Alzheimer's disease, early diagnosis remains a critical unmet need. Conventional diagnostic tools, including neuroimaging and cerebrospinal fluid biomarkers, are invasive, expensive or limited to pathological detection. Cognitive assessments are often administered after patients have started experiencing symptoms of cognitive decline.

Emerging evidence indicates a link between physical and cognitive abilities, with impairments in motor skills often occurring before cognitive decline in later years. This suggests that a decrease in physical ability might be an early indicator of future cognitive deterioration and an increased risk of dementia.^11^ Nonetheless, motor dysfunction in experimental models of Alzheimer's disease has not been thoroughly investigated.^12-14^

A better understanding of the disease to develop new diagnostic tools to reveal early signs of Alzheimer's disease is a clinical imperative that requires urgent solutions. From a diagnostic standpoint, while structural neocortical alterations are evident in the moderate stage of Alzheimer's disease, impairment of functional cortical connectivity and altered neuroplasticity can be detected in the preclinical and prodromal phases of Alzheimer's disease.^1,15^ In this context, EEG has emerged as a promising non-invasive technique for identifying early functional changes in brain activity associated with Alzheimer's disease. EEG-based biomarkers such as alterations in spectral power, coherence and complexity have been proposed as potential indicators of preclinical cognitive impairment and neuroinflammation.^16,17^

In the present study, we took advantage of the murine model of sporadic Alzheimer's disease induced by HSV-1 infection and recurrent reactivations to investigate early EEG alterations that are potentially predictive of Alzheimer's disease–like pathology. Specifically, C57BL/6 wild-type mice underwent two cycles of HSV-1 reactivation by thermal stress (2×TS, exhibiting mild memory impairment) or seven reactivations (7×TS, exhibiting irreversible cognitive deficits and Alzheimer's disease–like phenotype).^7,8,18^ Disease progression in this model has been previously documented^7,8^ and was monitored using canonical cognitive tests for memory impairments and molecular and immunohistochemical approaches for Alzheimer's disease hallmarks at the tissue level, particularly focusing on the frontal cortex (FC), controlling executive and motor functions.

This study addressed previously unexplored motor function impairment by characterizing the impact of the disease on grip strength and motor coordination. By combining behavioural analyses with EEG-like local field potential (LFP) recordings, we aimed to identify EEG signatures that may serve as early biomarkers of disease vulnerability to be translated into clinical applications.

## Materials and methods

### Ethics approval and consent to participate

All experiments and animal procedures were approved by the Catholic University Ethics Committee and were in line with the Italian (Ministry of Health guidelines, Legislative Decree No. 116/1992) and European Union (Directive No. 86/609/EEC) legislation on animal procedures (Animal protocol #17-43, approved by the Ministry of Health, Number 747/2022-PR 2022/12/12). All efforts were made to limit the number of animals used and minimize suffering.

For the human study component, all procedures were conducted in accordance with the Declaration of Helsinki and approved by the Institutional Ethical Committee (Prot. PG/2023/5172). Consent forms were previously signed by either the patients themselves or their next of kin.

### Animals

A total of 126 wild-type C57BL/6JRj male mice were used for this study. The mice were housed under standard animal housing conditions with a 12-h light/dark cycle, with constant humidity (60–75%), controlled ambient temperature (19–22°C) and food and water provided *ad libitum*.

### Participants

Male individuals with a definite diagnosis of Alzheimer's disease or mild cognitive impairment (MCI) were included in the study. Age-matched male healthy subjects (HS) were required to be aged 65–90 years with no medical, physical or cognitive conditions that might interfere with functional assessments.

### Animal model and experimental design

The experiments were designed in line with the ARRIVE guidelines. The HSV-1 Alzheimer's disease–like mouse model has been previously studied and well characterized.^7,8,18^ In particular, De Chiara *et al*.^7^ demonstrated that repeated reactivations of latent HSV-1 in the brain lead to the progressive development of Alzheimer's disease–like phenotype and cognitive decline. After infection and several cycles of TS-induced viral reactivation, HSV-1 spreads and actively replicates in different brain regions, particularly the neocortex and hippocampus, which are primarily affected in Alzheimer's disease. These reactivations cause the accumulation of key Alzheimer's disease molecular hallmarks, including hyperphosphorylated Tau, Aβ and markers of neuroinflammation, such as astrogliosis and increased levels of interleukin-1β and interleukin-6. The severity of these biochemical alterations and cognitive impairment correlates with the number of TS-inducing virus reactivations.^7^ Indeed, the authors showed that mice that were infected with HSV-1 but not subjected to TS (i.e. HSV-1 reactivation) did not manifest cognitive decline, confirming that viral reactivations, rather than initial infection or latency, are the key drivers of neuroinflammation and neurodegeneration.^7^ Based on this already documented evidence, we selected 2×TS and 7×TS as readouts of mild and severe stages, respectively, of the virus-induced disease, and we did not enrol a control group of mice infected with HSV-1 but not reactivated by TS. Instead, we included mock-infected mice (i.e. mice receiving vehicle inoculum and subjected to two or seven reactivations by TS) as controls to ensure that the observed effects were driven by HSV-1 reactivation rather than a consequence of TS.^19,20^ Naïve mice, which did not undergo any viral inoculation or reactivation procedures, were also included as further controls to consider the possible effects of age on the development of an Alzheimer's disease–like phenotype. The HSV-1 groups received virus inoculum and two (2×TS) or seven (7×TS) reactivations by TS. The mice were anesthetized with ketamine (65 mg/kg), medetomidine (0.65 mg/kg) and then infected via inoculation of HSV-1, strain F, with 2 µl of culture medium containing 1 × 10^6^ pfu of the virus through labial scarification under Biosafety level 2 conditions as previously described.^7,18^ Virus production was performed in Vero cells as previously reported.^21^ The vehicle inoculum for the mock groups consisted of the supernatants of mock-infected Vero cells. In particular, the 2×TS groups (HSV-1 and mock) were enrolled at 6.5 months of age, whereas the 7×TS groups were enrolled at 1.5 months of age such that at the time of LFP recordings, all the animals were of the same age. Therefore, the behavioural results after the second reactivation are presented separately for the 2×TS and 7×TS groups because at this time point, they had different ages, 9 and 4 months, respectively.

The 2×TS HSV-1 group received the first TS at 8 months and the second TS at 9 months of age. This group mimics the early stages of disease. The 7×TS HSV-1 group, which was infected with HSV-1 at approximately 1.5 months of age, underwent seven cycles of HSV-1 reactivation (1 cycle/month, starting at 3 months and continuing until 9 months). This group replicated an Alzheimer's disease–like phenotype. After receiving the vehicle inoculum, the corresponding sex- and age-matched controls, 2×TS mock and 7×TS mock mice, underwent two or seven TS, respectively. The reactivation procedure involved subjecting the mice to 15 min of TS obtained in an *ad hoc* environment at 43°C.^7,18^ The naïve group was enrolled at 1.5 months and sacrificed at the same age as the 2×TS and 7×TS groups (i.e. 9 months of age), serving as controls for both groups to disentangle the effects of age from those induced by viral reactivations.

**Figure 1:**
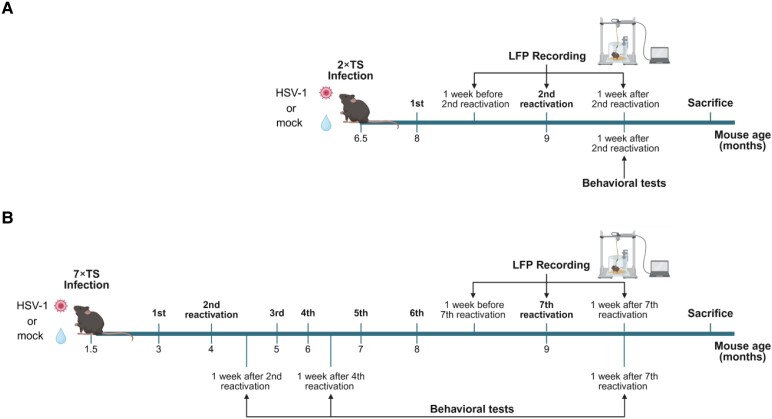
**Experimental design and timelines.** (**A**) 2×TS mice were infected with HSV-1 and subjected to two viral reactivations by TS, one each month, starting 1.5 months after infection. LFP recordings were performed 1 week before, 24 h and 1 week after the second reactivation. Behavioural tests were conducted during the second week after the second reactivation, followed by sacrifice and brain tissue explantation. (**B**) 7×TS mice were subjected to a total of seven viral reactivations by TS, one each month, starting 1.5 months after infection. Mock groups received vehicle inoculum. LFP recordings were performed 1 week before, 24 h and 1 week after the seventh reactivation. Behavioural assessments were performed in the week following the last LFP recordings. The animals were sacrificed at the end of the behavioural testing, and their brains were processed for immunofluorescence, molecular and morphological analyses. The figure was created in BioRender. Grassi, C. (2026) https://BioRender.com/ou1s01o.

As shown in Fig. 1, behavioural tests were conducted starting 1 week after the second reactivation in 2×TS mice and after the second, fourth and seventh reactivations in 7×TS and age-matched naïve mice. The animals were subjected to motor tasks to evaluate forelimb force and coordination and to cognitive tests to assess recognition and spatial memory. All behavioural tests were conducted after accurately evaluating the animals’ health status.

All mice underwent three LFP sessions: the first session was performed 1 week before the last reactivation, the second session was performed 24 h after reactivation and the last recording was performed 1 week after reactivation before the behavioural assessment (Fig. 1).

### Behavioural tests

The 2×TS group comprised 25 mock mice and 23 HSV-1 mice that underwent all behavioural tests. The 7×TS group included 15 naïve, 29 mock and 34 HSV-1 mice. The first pilot cohort of 7×TS mice (7 naïve, 14 mock and 14 HSV-1) performed all behavioural tasks after the second reactivation and after the seventh reactivation. A cohort of eight naïve mice underwent Novel Object Recognition (NOR), Y-maze, grid walking and grip strength tests after the seventh reactivation. Another cohort of 7×TS mice (15 mock and 20 HSV-1) underwent NOR after the second reactivation but not the Y-maze test because we did not observe impairment in the first cohort in this test. This cohort underwent all behavioural tests after the seventh reactivation. Additionally, these animals underwent a grip strength test starting from the second reactivation and after each reactivation to identify the onset of motor deficits. The same animals underwent the grid walking test at the onset of grip strength deficits, that is, the fourth reactivation. Motor and cognitive tasks were performed according to previously published protocols.^22-27^ Details are provided in the Supplementary Materials.

### Electrode implantation and LFP recordings

Two weeks before the second reactivation in 2×TS and the seventh reactivation in 7×TS mice, the animals underwent a surgical procedure for chronic electrode implantation. Recording electrodes were positioned over the FC, primary motor and somatosensory cortices of both sides.^26,27^ Data were acquired using the CerePlex Direct system (Blackrock Microsystem); LFP data were processed in MATLAB using scripts based on EEGLAB toolbox.^26,27^ In particular, the functional coupling of the LFP rhythms quantified using magnitude-squared coherence (MSCoh) for all combinations of electrode pairs, namely, the total magnitude-squared coherence (TotCoh), as detailed in the Supplementary Materials.^26-29^

### Immunofluorescence, molecular and morphological analyses

Immunofluorescence for glial fibrillary acidic protein (GFAP), Western immunoblotting for phosphorylated Tau at serine 199 (pTau^Ser199^) and Golgi-Cox staining were performed according to manufacturer’s instructions as detailed in the Supplementary Materials.

### Motor function assessment in human subjects

Handgrip strength was measured using a handgrip dynamometer (G200, Biometrics LTD, Newport, UK) over three 5-s isometric strength trials, with 1 min rest. The best absolute grip strength generated by each participant was recorded. The four-square step test (4SST) was employed as the closest proxy for the grid walking test to evaluate dynamic stability and coordination.^30^ A cross (plus sign) was drawn on the floor, and the participant was instructed to move clockwise from one square to the next, placing both feet in each square until they returned to the starting position. The test began when one foot touched the first square and ended when both feet touched the last square. The subject was instructed to look straight ahead and not touch the cross during the trial; otherwise, it was reported as an error. Two trials were performed, and the number of subjects who did not complete the 4SST correctly for each group was recorded.

### Statistical analysis

The sample size was determined with GPower 3.1.9.4 software considering the mean ± standard deviation of two groups, on the basis of the results of prior pilot datasets or studies, including our own, using similar methods or paradigms and considering a power of 80%, a confidence interval of 95% and a Type I error of 0.05 with two tails.

The data were analysed using GraphPad Prism or SigmaPlot 14.0 software and are expressed as the mean ± standard error of the means (SEM); a *P*-value <0.05 was considered statistically significant. The data were first tested for equal variance and normality (Shapiro‒Wilk test).

The behavioural results were analysed using unpaired two-tailed Student’s *t*-test or one-way ANOVA followed by a Bonferroni *post hoc* correction. Two-way repeated measures (RM) ANOVA (repetition factor: number of viral reactivations by TS) followed by a Bonferroni *post hoc* correction were used to analyse grip strength after each viral reactivation. Immunofluorescence and western blot data were analysed using an unpaired two-tailed Student’s *t*-test. Spine density results were analysed using a linear mixed-effects model (MATLAB) with experimental groups as a fixed effect and animal as a random effect to account for multiple neurons sampled per animal.

For the analysis of the LFP data, a two-way RM ANOVA was performed to assess differences in TotCoh across experimental groups (mock, 2×TS HSV-1) and frequency bands (delta, theta, alpha 1, alpha 2, beta 1, beta 2 and gamma), treating frequency bands as a repeated factor per subject. Only data from the third LFP session were used, namely, data recorded 1 week after reactivation. *Post hoc* comparisons were conducted using Duncan’s test. Additionally, Pearson’s correlation analysis was used to evaluate the relationship between TotCoh within each frequency band and grip strength, NOR test and Y-maze redouts, pooling data from all mice (2×TS mock, 7×TS mock, 2×TS HSV-1 and 7×TS HSV-1) into a single group.

With respect to human subjects, handgrip data were analysed by one-way ANOVA (group) with a Bonferroni-corrected *post hoc* test. The number of subjects who did not complete the 4SST correctly was used to calculate odds ratios (completed/not completed in each group) and was tested statistically using the likelihood ratio.

## Results

### 2×TS HSV-1 mice exhibit memory deficits that worsen in 7×TS HSV-1 mice

Based on previously published studies,^7,8,18^ we selected the 2×TS and 7×TS paradigms as representative of early (mild) and advanced (severe) stages of the HSV-1-induced Alzheimer’s disease–like phenotype, respectively.

Before conducting each behavioural test, we examined the general state of health of the mice, particularly after each TS.

Accurate inspection of the animals did not reveal any signs of hunching, paralysis, dehydration after each reactivation or abnormal behaviour reflecting signs of sickness. Moreover, no significant differences in body weight, used as a further parameter to evaluate animal welfare, were observed between the HSV-1 and mock groups after each reactivation (Supplementary Fig. 1).

**Figure 2:**
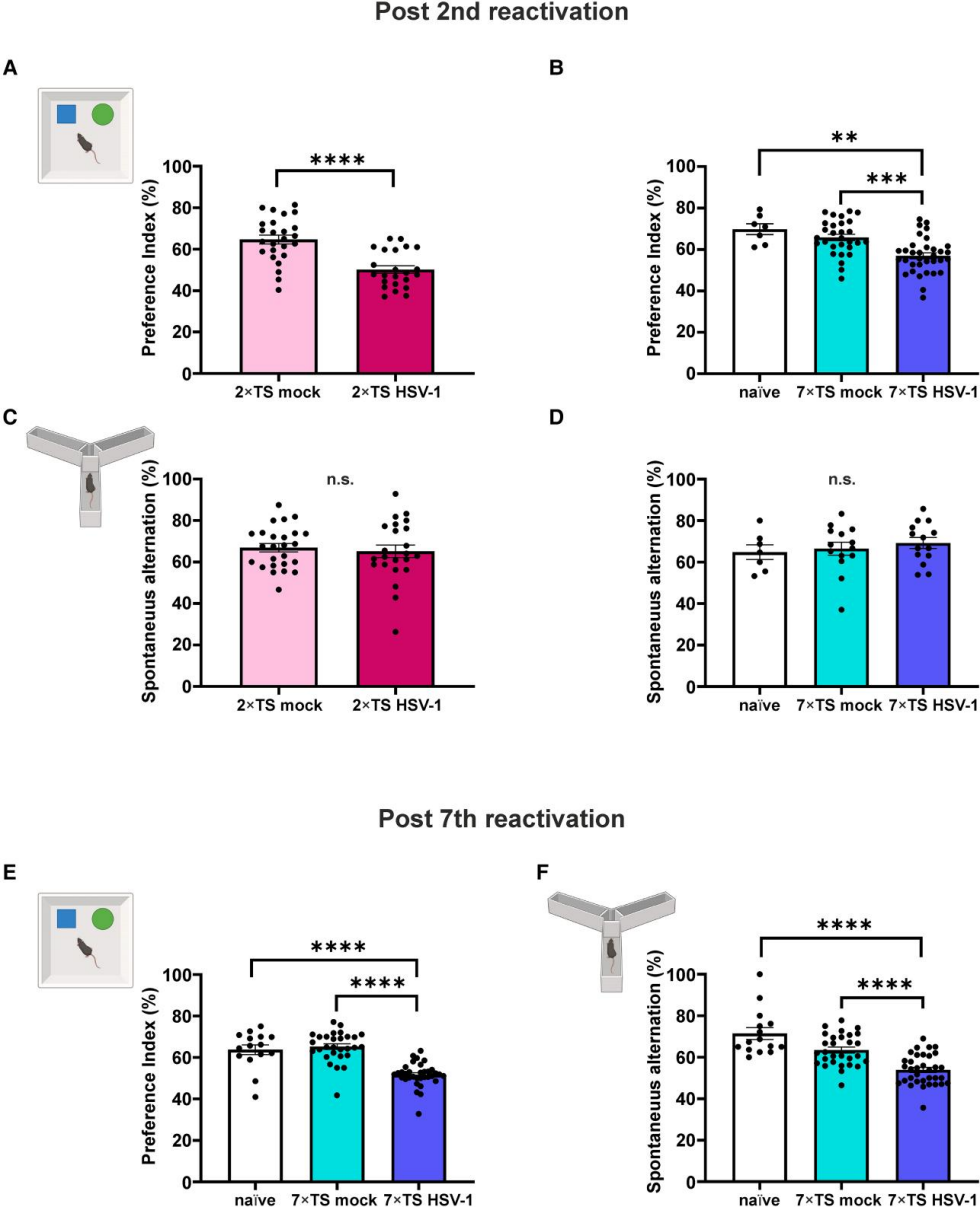
**Cognitive performances of 2×TS and 7×TS HSV-1, mock and naïve mice.** NOR test (2×TS mock: *n* = 25; 2×TS HSV-1: *n* = 23; naïve: *n* = 7; 7×TS mock: *n* = 29; 7×TS HSV-1: *n* = 34) revealed a significant impairment in recognition memory both in 2×TS HSV-1 (**A**) and 7×TS HSV-1 mice (**B**) compared to their respective mock groups and naïve mice after the second reactivation. (**C**, **D**) Y-maze test showed no significant differences in spontaneous alternations post second reactivation (2×TS mock: *n* = 25; 2×TS HSV-1: *n* = 23; naïve: *n* = 7; 7×TS mock: *n* = 14; 7×TS HSV-1: *n* = 14). After the seventh reactivation, 7×TS HSV-1 mice (naïve: *n* = 15; 7×TS mock: *n* = 29; 7×TS HSV-1: *n* = 34) showed cognitive impairment in NOR (**E**) and in Y-maze tests (**F**). Data are presented as mean ± SEM. **P < 0.01; ***P < 0.001; ****P < 0.0001; one-way ANOVA, Bonferroni *post hoc*; n.s., not significant. Each dot represents an individual animal. The illustrations are created in BioRender. Grassi, C. (2026) https://BioRender.com/it268ml.

Both the 2×TS and 7×TS groups were tested with a battery of cognitive tests to assess memory function after the second reactivation (note that the 2×TS and 7×TS groups differed only by the age at which they underwent infection and consequently the second reactivation, namely, 9 and 4 months, respectively). The results demonstrated that all HSV-1 mice showed deficits in recognition memory after the second TS, as indicated by the significantly lower preference index value in the NOR test compared with the respective mock groups. In particular, preference index was 50.2 ± 1.8% in 2×TS HSV-1 mice (*n* = 23) and 64.7 ± 2.2% in 2×TS mock mice (*n* = 25; *P* < 0.0001; Student’s *t*-test; Fig. 2A).

Similarly, following the second reactivation, the 7×TS HSV-1 group showed a significantly lower preference index compared to 7×TS mock and naïve mice [57.0 ± 1.5%, 7×TS HSV-1 (*n* = 34) versus 65.8 ± 1.6%, 7×TS mock (*n* = 29); *P* < 0.001; and versus 69.9 ± 2.6%, naïve (*n* = 7); *P* < 0.01; one-way ANOVA, Bonferroni *post hoc*; *F*(2, 67) = 12.25; Fig. 2B]. No differences were observed between the 7×TS mock group and the naïve group (*P* = 0.763; Fig. 2B).

After the second reactivation, no impairment was observed in spatial memory in either the 2×TS or 7×TS groups, as revealed by a similar percentage of spontaneous alternations in the Y-maze tests. In particular, in the 2×TS groups the percentage of spontaneous alternations was 65.2 ± 3.1% in HSV-1 mice (*n* = 23) and 66.9 ± 2.0% in mock mice (*n* = 25; *P* = 0.629; Student’s *t*-test; Fig. 2C). In 7×TS groups, it was 69.2 ± 2.6% in HSV-1 mice (*n* = 14), 66.5 ± 3.1% in mock mice (*n* = 14) and 64.8 ± 3.6% naïve mice [*n* = 7; *F*(2, 32) = 0.4634; *P* = 0.6333; one-way ANOVA; Fig. 2D.

After the seventh reactivation, the impairment in recognition memory persisted, as revealed by lower preference index of 7×TS HSV-1 mice (51.7 ± 1.0%; *n* = 34) compared to 7×TS mock (65.2 ± 1.3%; *n* = 29; *P* < 0.0001) and to naïve mice [63.8 ± 2.3%; *n* = 15; *P* < 0.0001; *F*(2, 75) = 34.12; one-way ANOVA, Bonferroni *post hoc*; Fig. 2E].

Notably, in the same mice, a spatial memory deficit emerged, as revealed by a decreased percentage of spontaneous alternation in the Y-maze in 7×TS HSV-1 mice compared to 7×TS mock and naïve animals [53.9 ± 1.3%, 7×TS HSV-1 versus 63.5 ± 1.4%, 7×TS mock: *P* < 0.0001; and versus 71.5 ± 2.9%, naïve; *P* < 0.0001; *F*(2, 75) = 25.94; one-way ANOVA, Bonferroni *post hoc*; Fig. 2F], in keeping with the worsening of cognitive dysfunction following repeated reactivations.^8,18^

### HSV-1 mice exhibit motor deficits with an onset after the fourth viral reactivation

**Figure 3:**
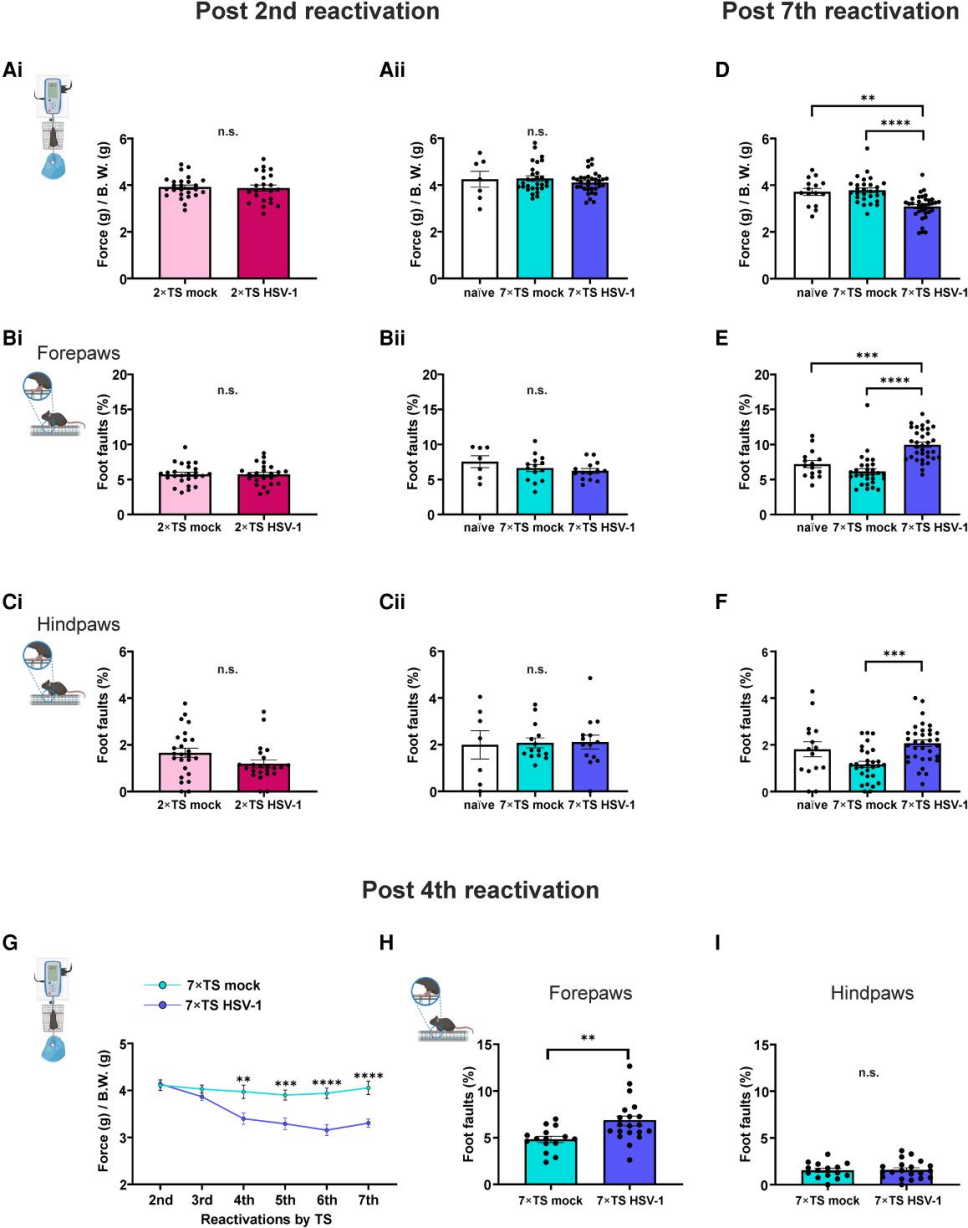
**Characterization of motor performance in 2×TS and 7×TS HSV-1, mock and naïve mice.** (**Ai–Ci**) After the second reactivation (2×TS mock: *n* = 25; 2×TS HSV-1: *n* = 23), no impairments were observed in the 2×TS experimental groups in the grip strength test (**Ai**) or grid walking test (**Bi**, **Ci**). The same picture emerged for the 7×TS and naïve groups (**Aii–Cii**): panel **Aii** (naïve: *n* = 7; 7×TS mock: *n* = 29; 7×TS HSV-1: *n* = 34); panels **Bii** and Cii (naïve: *n* = 7; 7×TS mock: *n* = 14; 7×TS HSV-1: *n* = 14). Conversely, after the seventh reactivation (naïve: *n* = 15; 7×TS mock: *n* = 29; 7×TS HSV-1: *n* = 34), HSV-1 mice performed worse than mock and naïve mice did in both the grip strength (**D**) and grid walking tests (**E**, **F**). (**G**) Assessment of grip strength from the second to the seventh reactivation revealed the onset of deficits after the fourth reactivation in HSV-1 mice (7×TS mock: *n* = 15; 7×TS HSV-1: *n* = 20). Compared with the 7×TS mock control mice, after the fourth reactivation (7×TS mock: *n* = 15; 7×TS HSV-1: *n* = 20), the HSV-1 mice presented an increased percentage of foot faults with their forelimbs (**H**) but not their hindlimbs (**I**). The data are presented as the mean ± SEM. **P < 0.01; ***P < 0.001; ****P < 0.0001. One-way ANOVA, Bonferroni *post hoc* correction (**Aii–Cii**; **D–F**), RM two-way ANOVA, Bonferroni *post hoc* correction (**G**) and Student’s *t*-test (**Ai–Ci**; **HI**). n.s., not significant; B.W., body weight. Each dot represents an individual animal. The illustrations are created in BioRender. Grassi, C. (2026) https://BioRender.com/8p69e5b.

Motor performance was evaluated in naïve mice and in 2×TS, 7×TS, following the second and the seventh TS. After the second reactivation, no significant differences were detected between the 2×TS HSV-1 (*n* = 23 mice) and 2×TS mock groups (*n* = 25 mice) either in grip strength (forelimb force: 3.9 ± 0.1 versus 3.9 ± 0.1; *P* = 0.815; Student’s *t*-test; Fig. 3Ai) or in the grid walking tests (percentage of forepaw faults: 5.7 ± 0.3% versus 5.7 ± 0.3%; *P* = 0.95; Fig. 3Bi; percentage of hindpaw faults: 1.2 ± 0.2% versus 1.7 ± 0.2%; *P* = 0.1; Student’s *t*-test; Fig. 3Ci).

Similarly, the 7×TS and naïve groups did not show any significant motor deficits after the second reactivation. In particular, the forelimb force [(g)/body weight (g)] was 4.1 ± 0.1 in the 7×TS HSV-1 group (*n* = 34), 4.3 ± 0.1 in the 7×TS mock group (*n* = 29) and 4.2 ± 0.3 in the naïve group (*n* = 7)*(P* = 0.4764; one-way ANOVA; Fig. 3Aii). No differences among groups were detected in the grid walking test [percentage of forepaw faults: 6.2 ± 0.4% in 7×TS HSV-1 (*n* = 14); 6.6 ± 0.5% in 7×TS mock (*n* = 14); 7.5 ± 0.8% in naïve (*n* = 7); *F*(2, 32) = 1.313; *P* = 0.2831; Fig. 3Bii; percentage of hindpaw faults: 2.1 ± 0.3% 7×TS HSV-1; 2.1 ± 0.2% 7×TS mock; 2.0 ± 0.6% naïve; *F*(2, 32) = 0.02691; *P* = 0.9735; one-way ANOVA; Fig. 3Cii].

Interestingly, after the seventh reactivation, the 7×TS HSV-1 mice presented motor deficits that were not observed in the 7×TS mock and naïve mice, as revealed by decreased forelimb strength [3.1 ± 0.1 (*n* = 34 mice) versus 3.8 ± 0.1, 7×TS mock mice (*n* = 29); *P* < 0.0001 and versus 3.7 ± 0.1 naïve (*n* = 15); *P* = 0.001; *F*(2, 75) = 14.95; one-way ANOVA, Bonferroni *post hoc*; Fig. 3D] and increased percentage of forepaw faults [9.9 ± 0.4% versus 6.1 ± 0.4% 7×TS mock; *P* < 0.0001; versus 7.20 ± 0.5% naïve; *P* < 0.001; *F*(2, 75) = 24.47; one-way ANOVA, Bonferroni *post hoc*; Fig. 3E] and of hindpaw faults [2.1 ± 0.1% versus 1.2 ± 0.1% 7×TS mock; *F*(2, 75) = 8.370; *P* < 0.001; one-way ANOVA, Bonferroni *post hoc*; Fig. 3F].

Having demonstrated the motor impairments in the more severe stage of the pathology, we then characterized the onset of grip strength deficit in a subgroup of 7×TS mice by performing the test 48 h after each reactivation, starting from the second reactivation.

Statistical analysis via two-way RM ANOVA (repetition factor: number of viral reactivations) revealed a main effect of group [*F*(1, 33) = 29.82; *P* < 0.0001] and a main effect of the number of reactivations [*F*(5, 165) = 9.763; *P* < 0.0001], with a significant group × reactivation interaction [*F*(5, 165) = 5.296; *P* = 0.0002]. Bonferroni *post hoc* analyses revealed significant deficits in 7×TS HSV-1 mice (*n* = 20) compared with mock mice (*n* = 15), which emerged at the fourth reactivation and remained almost unchanged across the subsequent TS (fourth: 3.40 ± 0.12 versus 4.0 ± 0.14; *P* = 0.002; fifth 3.29 ± 0.13 versus 3.90 ± 0.11; *P* = 0.0008; sixth: 3.15 ± 0.11 versus 3.94 ± 0.11; *P* < 0.0001; seventh: 3.30 ± 0.09 versus 4.05 ± 0.14; *P* < 0.0001; Fig. 3G).

Having identified the onset of grip strength deficits, we also tested the same animals in the grid walking test after the fourth reactivation and observed increased foot faults with forepaws in 7×TS HSV-1 mice compared with those in 7×TS mock mice (6.8 ± 0.5% versus 4.8 ± 0.3; *P* < 0.01; Student’s *t*-test; Fig. 3H), but no differences in foot fault percentage were detected with hindpaws compared with those in the mock group (1.6 ± 0.2% versus 1.5 ± 0.2; *P* = 0.86; Student’s *t*-test; Fig. 3I).

### HSV-1 mice show stage-dependent astrocytic activation and expression of Alzheimer's disease hallmark

Consistent with previous studies^7,8,18^ and our behavioural evidence, immunofluorescence and western blot analyses revealed the stage-dependent appearance of Alzheimer's disease hallmarks. Specifically, we assessed the expression of GFAP as a marker of astrocytic activation and pTau accumulation, focusing on FC, which is of particular interest, given its involvement in motor control. Detailed analyses of the neuroinflammatory profile and Alzheimer's disease protein accumulation (pTau and β-amyloid) in different brain areas, such as the hippocampus, were beyond the scope of this study and have been thoroughly investigated in previous^7,8,18^ and more recent studies.^31^

**Figure 4:**
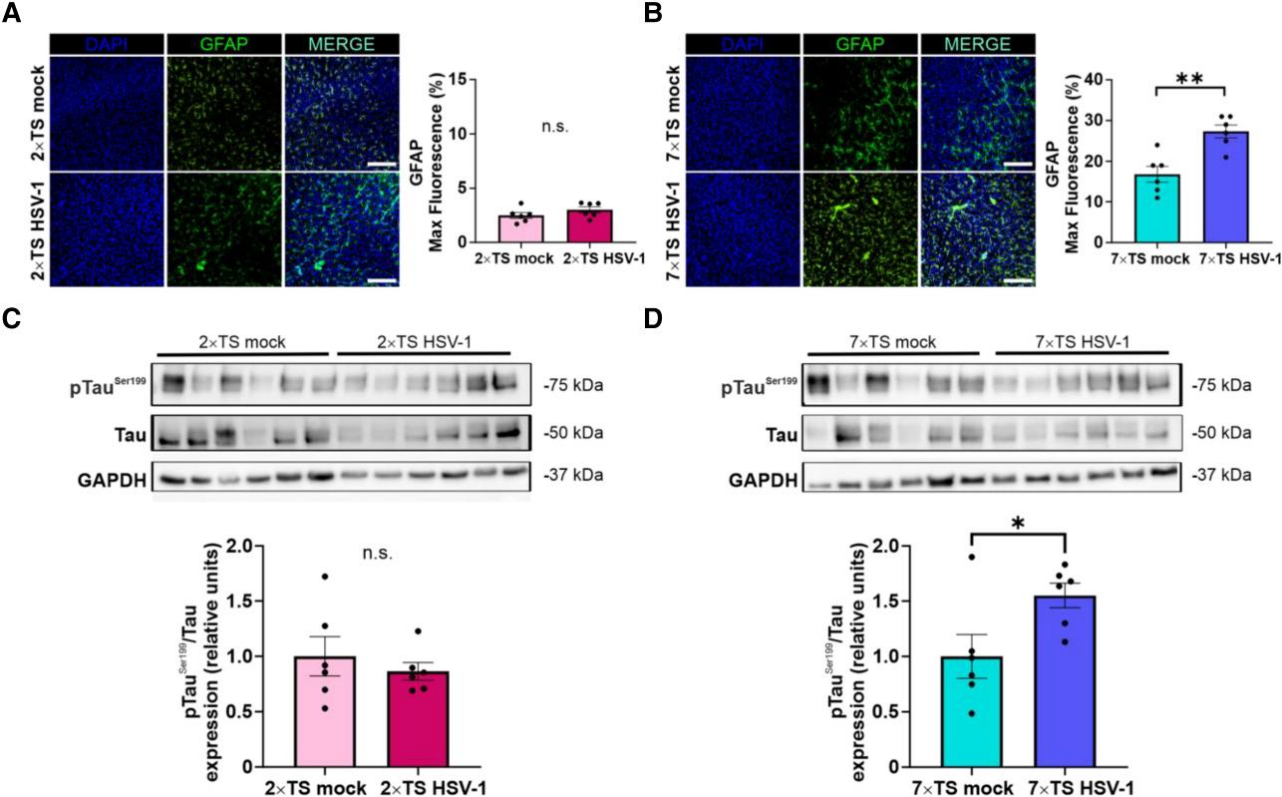
**Astrocytic activation and pTau^Ser199^ expression in 2×TS and 7×TS HSV-1 and mock mice.** (**A**) Quantification of GFAP fluorescence intensities in the FC revealed no significant differences between the 2×TS HSV-1 (*n* = 6) and 2×TS mock (*n* = 6) groups. Representative immunofluorescence images show GFAP^+^ astrocytes (green) and DAPI^+^ cell nuclei (blue) in the FC of 2×TS HSV-1 and 2×TS mock mice. (**B**) A significant increase of GFAP signal was observed in 7×TS HSV-1 mice (*n* = 6) compared to 7×TS mock (*n* = 6) in the FC. Representative immunofluorescence images show GFAP^+^ astrocytes (green) and DAPI^+^ cell nuclei (blue) in the FC of 7×TS HSV-1 and 7×TS mock mice. Scale bar: 100μm. (**C**) Representative western immunoblots and band densitometry showing no difference in the level of expression of pTau^Ser199^ between 2×TS HSV-1 (*n* = 6) and 2×TS mock (*n* = 6) groups. Corresponding uncropped western blots are shown in Supplementary Fig. 2A. (**D**) Western immunoblots and band densitometry showed increased levels of pTau^Ser199^ in 7×TS HSV-1 mice (*n* = 6) compared to 7×TS mock mice (*n* = 6). Corresponding uncropped western blots are shown in Supplementary Fig. 2B. The data are presented as the mean ± SEM. *P < 0.05; **P < 0.01; Student’s *t*-test, n.s., not significant. Each dot represents an individual animal.

In the 2×TS group, no significant differences in GFAP fluorescence intensity (expressed as the percentage of threshold area occupied by positive staining) were observed between HSV-1 and mock-infected mice in the FC (3.0 ± 0.3% versus 2.5± 0.3%; *P* = 0.2059; Student’s *t*-test; Fig. 4A).

Conversely, GFAP fluorescence intensity was significantly greater in 7×TS HSV-1 mice than in the 7×TS mock in the FC (27.3 ± 1.6% versus 16.8 ± 1.9%; *P* = 0.0019; Student’s *t*-test; Fig. 4B). These data indicate that a marked glial response in FC is evident at later stages of disease progression.

To further validate the model in the context of Alzheimer's disease–like phenotype, we examined the expression of pTau^Ser199^/Tau ratio in the FC of HSV-1 and mock mice. At the early 2×TS stage, pTau^Ser199^ levels were comparable between the HSV-1 and mock groups (*P* = 0.5012, *n* = 6 mice/group; Student’s *t*-test; Fig. 4C and Supplementary Fig. 2A).

However, in the advanced 7×TS stage, there was a significant increase in pTau^Ser199^ in the HSV-1 group compared to the mock group (+55%; *P* = 0.035; *n* = 6 mice/group; Student’s *t*-test; Fig. 4D, and Supplementary Fig. 2B).

Collectively, these neuropathological findings indicate stage-dependent development of the astrogliosis and Alzheimer's disease hallmarks, recapitulating the gradual development of neuropathological features of Alzheimer's disease.

### Cortical connectivity alterations in 2×TS HSV-1 significantly correlate only with grip strength

**Figure 5:**
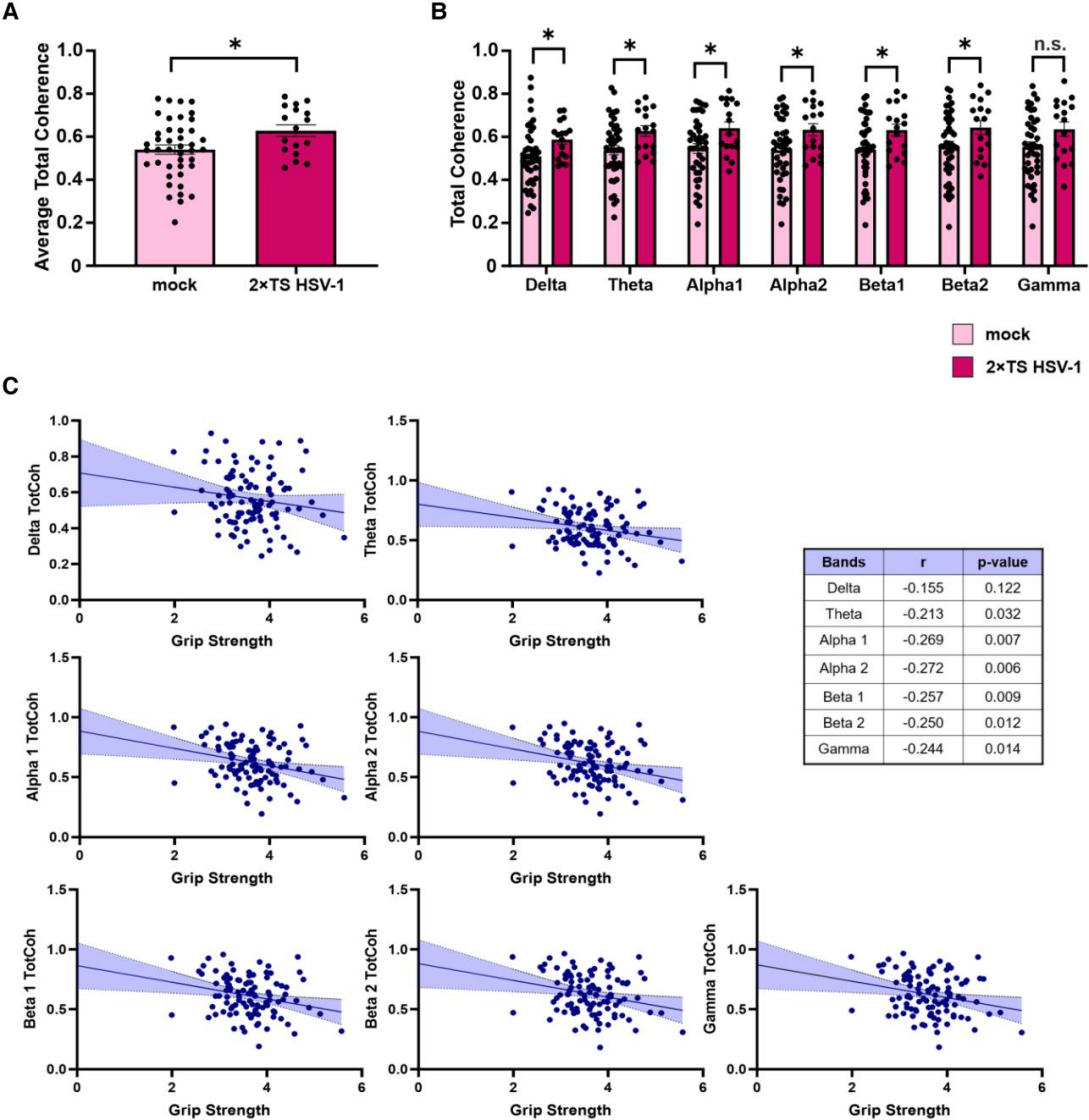
**Cortical connectivity changes in 2×TS HSV-1-treated mice compared with mock mice.** (**A**) Group differences in TotCoh across frequency bands. Overall, TotCoh was significantly greater in the 2×TS HSV-1 group (*n* = 17) than in the mock group (*n* = 42), as revealed by two-way RM ANOVA, with group and frequency band as factors [*F*(1, 57) = 5.34, *P* = 0.024]. (**B**) This effect was consistently observed across multiple frequency bands, with significant group differences in delta (*P* = 0.042), theta (*P* = 0.020), alpha 1 (*P* = 0.021), alpha 2 (*P* = 0.021), beta 1 (*P* = 0.026), beta 2 (*P* = 0.042) and a trend in the gamma band (*P* = 0.057). (**C**) Pearson’s correlation analysis between TotCoh, computed from LFP signals across different frequency bands (delta, theta, alpha 1, alpha 2, beta 1, beta 2 and gamma), and grip strength. The table displays the correlation coefficients (*r*) and corresponding *P* values for each frequency band. Significant negative correlations were observed between grip strength and TotCoh in the theta (*P* = 0.032), alpha 1 (*P* = 0.007), alpha 2 (*P* = 0.006), beta 1 (*P* = 0.009), beta 2 (*P* = 0.012) and gamma (*P* = 0.014) bands, indicating that greater coherence in these bands is associated with lower grip strength. The data are presented as the mean ± SEM. *P  *<* 0.05; Student’s *t*-test, n.s., not significant (**A**, **B**). Each data point represents a different animal.

To investigate the pattern of TotCoh, a two-way RM ANOVA was conducted considering group (mock, 2×TS HSV-1) and frequency bands (delta, theta, alpha 1, alpha 2, beta 1, beta 2 and gamma) as factors, with frequency bands treated as a within-subject variable to account for biological variability. A statistically significant main effect of group was observed, with the 2×TS HSV-1 group showing higher TotCoh values than the mock group did [*F*(1, 57) = 5.34, *P* = 0.024; Fig. 5A]. This group effect was evident across multiple frequency bands, with significant differences in delta (*P* = 0.042), theta (*P* = 0.020), alpha 1 (*P* = 0.021), alpha 2 (*P* = 0.021), beta 1 (*P* = 0.026) and beta 2 (*P* = 0.042) values and a trend towards significance in the gamma band (*P* = 0.057; Fig. 5B). These results suggest a general increase in functional connectivity (TotCoh) across frequency bands in the 2×TS HSV-1 group compared with the control group, which might reveal early network alterations in the Alzheimer's disease–like mouse model.

Notably, Pearson’s correlation analysis revealed statistically significant negative correlations between TotCoh and grip strength across several frequency bands (Fig. 5C). Specifically, significant negative correlations were detected in the theta (*r* = −0.213, *P* = 0.032), alpha 1 (*r* = −0.269, *P* = 0.007), alpha 2 (*r* = −0.272, *P* = 0.006), beta 1 (*r* = −0.257, *P* = 0.009), beta 2 (*r* = −0.250, *P* = 0.012) and gamma (*r* = −0.244, *P* = 0.014) bands. The delta band showed a non-significant trend (*r* = −0.155, *P* = 0.122). These findings indicate that greater coherence within these frequency bands is associated with lower grip strength.

Additionally, Pearson’s correlation analysis revealed no statistically significant relationships between TotCoh and NOR test performance across any frequency band. Specifically, no significant correlations were observed in the delta (*r* = −0.060, *P* = 0.551), theta (*r* = −0.044, *P* = 0.665), alpha 1 (*r* = −0.063, *P* = 0.533), alpha 2 (*r* = −0.050, *P* = 0.616), beta 1 (*r* = −0.037, *P* = 0.712), beta 2 (*r* = −0.005, *P* = 0.958) or gamma (*r* = 0.008, *P* = 0.936) bands. Similarly, no significant correlations were found between TotCoh and Y-maze spontaneous alternation across frequency bands, including delta (*r* = 0.192, *P* = 0.054), theta (*r* = 0.130, *P* = 0.195), alpha 1 (*r* = 0.080, *P* = 0.422), alpha 2 (*r* = 0.074, *P* = 0.465), beta 1 (*r* = 0.087, *P* = 0.389), beta 2 (*r* = 0.076, *P* = 0.399) and gamma (*r* = 0.008, *P* = 0.449) bands. In summary, these results indicate that TotCoh is specifically associated with motor function, as reflected by its significant correlation with grip strength, but not with cognitive performance measures obtained with the NOR or Y-maze tests.

### Morphological analysis reveals early dendritic spine changes in the motor cortex of HSV-1 mice

**Figure 6:**
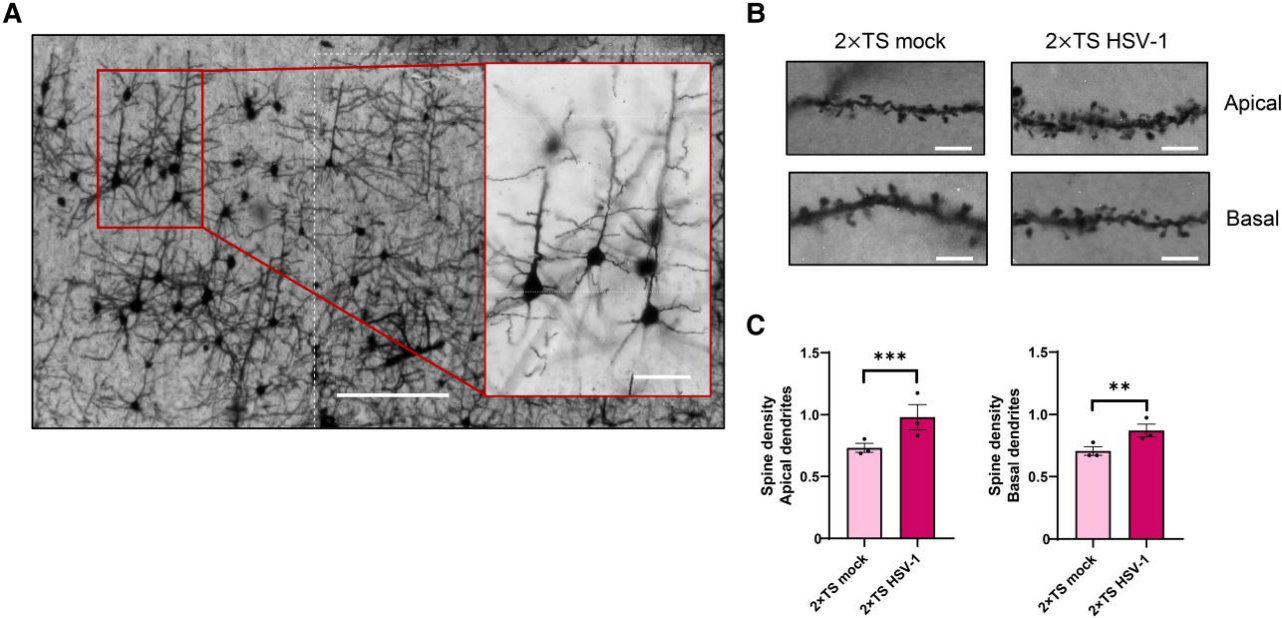
**Morphological analysis of dendritic spine density in 2×TS HSV-1 and mock mice.** (**A**) Representative low- (4×; scale bar, 200 µm) and high-magnification (20×; scale bar, 50 µm) images of Golgi-Cox–stained pyramidal neurons in cortical Layers II/III of the primary motor cortex from a 2×TS mock mouse. (**B**) Representative high-magnification images (100×) of the dendritic segments used for spine density analysis in 2×TS mock and HSV-1 mice (scale bar: 5 µm). (**C**) Bar graphs showing increased spine density in the apical and basal dendrites of pyramidal neurons in 2×TS HSV-1 mice compared with mock mice (*n* = 3 mice/group, *n* = 10 neurons/mouse). The data are presented as mean ± SEM calculated across animals. **P < 0.01; ***P < 0.001. Statistical analysis was performed using a linear mixed-effects model. Each dot represents an individual animal.

Given the negative correlation between TotCoh and grip strength, we hypothesized that altered connectivity might result from alterations in synaptic contacts in the motor cortex. Golgi-Cox staining was then performed to analyse dendritic spine density in Layer II/III pyramidal neurons of the primary motor cortex in the 2×TS HSV-1 and 2×TS mock groups (*n* = 3 mice/group; *n* = 10 neurons/mouse; Fig. 6A).

Spine density was quantified separately on apical and basal dendrites. To account for the non-independence of repeated measurements obtained from the same animal, experimental group differences in apical and basal dendritic spine count were assessed using a linear mixed-effects model with mouse as a random effect. In the apical compartment, the model revealed a significant effect of group, indicating that 2×TS HSV-1 mice exhibited significantly higher apical spine values compared to mock mice [β = −0.247, 95% CI: −0.371, −0.124, *F*(1,58) = 16.14, *P* = 1.7 × 10^−4^, Fig. 6B and C].

Similarly, basal dendritic spine measurements revealed a significant effect of group, indicating that mock mice exhibited significantly lower basal dendritic spine values compared to HSV-treated mice [β = −0.172, 95% CI: −0.268, −0.076, *F*(1,58) = 12.95, *P* = 6.6 × 10^−4^, Fig. 6B and C]. It might be speculated that the increased spine density in both the apical and basal compartments of motor cortex pyramidal neurons reflect maladaptive synaptic remodelling processes occurring in the early stages of the pathology, leading to altered connectivity preceding motor impairment.

### Early impairments in motor function are evident in MCI patients

To substantiate our experimental model and explore the applicability of our findings in clinical settings, we conducted a preliminary validation of our results in human participants concerning motor deficits. This involved testing HS, MCI and Alzheimer's disease subjects of comparable age (mean age: 74.76 ± 8.18 years; HS: 73.22 ± 7.29; MCI: 74.70 ± 7.37; Alzheimer's disease: 76.92 ± 10.15; *P* = 0.248) using motor tests analogous to those utilized in the experimental model, i.e. grip strength and 4SST.

**Figure 7:**
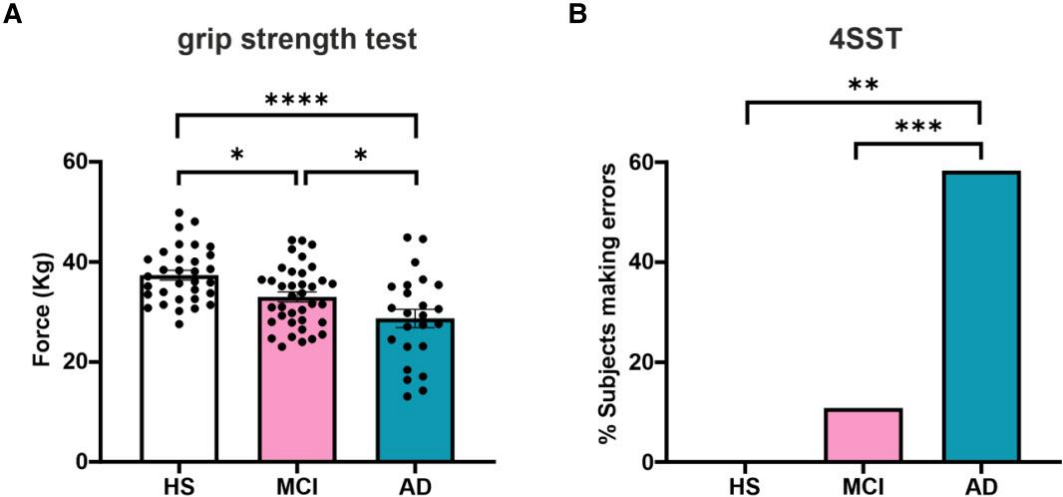
**Characterization of motor tests performed by human participants.** (**A**) Bar graph showing the results of the grip strength test (force: kg in the different groups of participants, HS, *n* = 32; MCI, *n* = 37; AD, *n* = 24). The data are presented as the mean ± SEM; one-way ANOVA, Bonferroni *post hoc* correction. (**B**) Bar graph showing the percentage of subjects who did not complete the 4SST correctly; the data were analysed via likelihood ratio tests. *P < 0.05; **P < 0.01; ***P < 0.001; ****P < 0.0001. Each dot represents an individual participant.

With respect to grip strength, significant differences were observed. Compared to HS (force: 37.36 ± 5.53 kg; *n* = 32), both MCI (*n* = 37; 33.03 ± 6.06 kg; −11.6% versus HS; *P* = 0.027) and Alzheimer's disease (*n* = 24; 28.72 ± 8.86 kg; −23.1% versus HS; *P* < 0.0001) generated significantly less strength. Moreover, MCI patients’ grip strength was significantly higher than Alzheimer's disease (+15%; *P* = 0.049; Fig. 7A).

Significant differences were also observed among groups in the 4SST, which served as a motor proxy for assessing dynamic stability and coordination: Alzheimer's disease patients made a greater number of errors than MCI (*P* = 0.0003) and HS (*P* = 0.002). Although the difference was not statistically significant, the MCI tended to make more errors than the HS did (*P* = 0.15; Fig. 7B).

## Discussion

Alzheimer's disease is a progressive neurodegenerative disorder characterized by cognitive decline and classical neuropathological features, including Aβ plaques and hyperphosphorylated Tau. While the molecular features of the advanced Alzheimer's disease stage are well described, the identification of reliable early biomarkers and the delineation of the events that initiate circuit dysfunction remain critical unmet challenges. In this study, we tracked the onset of behavioural dysfunctions and brain activity alterations, assessed by *in vivo* LFP recordings, in a mouse model of sporadic Alzheimer's disease based on HSV-1 infection and repeated reactivations by TS. We chose a model reproducing features of the sporadic Alzheimer's disease, because commercially available genetic models primarily replicate the much rarer familial form of Alzheimer's disease, which may not accurately reflect the complexity of the disease, as over 95% of Alzheimer's disease cases are sporadic. The HSV-1 model, instead, combines two of the many risk factors that have been implicated in Alzheimer's disease, namely, the viral infection and neuroinflammation, making the model relevant for research. The HSV-1 Alzheimer's disease–like mouse model has been previously characterized, showing that after infection and several cycles of TS-induced viral reactivation, HSV-1 spreads and actively replicates in different brain regions, particularly the neocortex and hippocampus, which are primarily affected by Alzheimer's disease.^7^ Repeated reactivations of latent HSV-1 in the brain lead to the progressive development of Alzheimer's disease–like pathology, causing the accumulation of hyperphosphorylated Tau, Aβ and neuroinflammation markers. The severity of these alterations correlates with reactivation frequency and cognitive decline.^7^ Moreover, previous studies have demonstrated that mice infected but not subjected to stress (and therefore not reactivated) did not show cognitive decline, confirming that viral reactivation, rather than initial infection or latency, is the key driver of neurodegeneration.^7^

In line with previous studies,^7,8,18^ we observed that repeated HSV-1 reactivations triggered cognitive impairments and Alzheimer's disease hallmarks, including Tau phosphorylation and astrogliosis in the cortex at later stages (i.e. in 7×TS HSV-1 mice). Although previous studies have reported direct effects of TS on the biochemical and functional features of the mouse brain,^19,20^ the inclusion of both mock and naïve control groups in our experimental design, neither of which exhibited functional deficits, effectively ruled out a major contribution of TS or age to the behavioural alterations observed under our experimental conditions. Selective impairment in recognition memory was observed after two viral reactivations in the absence of spatial memory or motor deficits, indicating early vulnerability of recognition circuits. The discrepancy with previous studies that reported impaired spatial memory in the Y-maze test after two viral reactivations^8^ might be attributed to greater individual variability at the initial stage of the pathology.

Here, we identified a novel finding in the field: consistent impairments in grip strength and coordination that emerged after the fourth viral reactivation and persisted over time, remaining stable throughout subsequent reactivations. This sustained impairment highlights the robustness and temporal consistency of the motor phenotype and strengthens its potential as an early marker. The temporally specific emergence of durable motor deficits in a mouse model of sporadic Alzheimer's disease represents the central novelty of our study. These motor alterations, which have not yet been described in HSV-1 models and have been studied in other murine Alzheimer's disease models in later stages,^12-14^ suggest the presence of intrinsic circuit-level vulnerabilities that precede structural degeneration.

Another major strength of our study is the identification of functional connectivity changes as early electrophysiological correlations of network vulnerability. EEG recordings revealed increased TotCoh levels in 2×TS HSV-1 mice, indicative of early network hyperconnectivity, which is consistent with evidence from human subjects, supporting the translational relevance of our findings. Previous studies have described altered EEG coherence patterns as early signatures of Alzheimer's disease–related network dysfunction,^15-17,32^ supporting the hypothesis that altered large-scale network synchrony may be one of the earliest measurable indicators of vulnerability to Alzheimer's disease.

This hypersynchrony, which we interpret as a marker of early circuit instability, was not merely observational, as it was negatively correlated with grip strength performance across multiple frequency bands, suggesting a direct relationship between altered cortical dynamics and subclinical motor decline. Similar hypersynchronous EEG patterns have been associated with early Alzheimer's disease and MCI stages in humans,^16,17^ further supporting the translational relevance of our findings.

These findings suggest that early hyperconnectivity, particularly within the alpha and beta bands, which are implicated in motor control, may reflect maladaptive network dynamics. The association between increased coherence and reduced motor performance supports the notion that aberrant large-scale synchrony may contribute to early functional decline. These findings further underscore the potential of cortical hyperconnectivity as an early relevant marker of vulnerability to Alzheimer's disease. No correlation was found between TotCoh and the NOR or Y-maze indices, further corroborating the relevance of motor readouts as functional correlates of brain connectivity changes.

Importantly, these electrophysiological and behavioural changes were accompanied by an increase in dendritic spine density in both the apical and basal dendrites of Layer II/III pyramidal neurons in the motor cortex. This finding is consistent with reports on early synaptic remodelling in neurodegenerative disease models.^33^ While this may reflect an initial attempt at compensatory plasticity, such remodelling could lead to maladaptive network organization, underlying the observed hypersynchrony and potentially contributing to the observed motor dysfunctions. These findings support the idea that motor alterations are not simply secondary to widespread neurodegeneration but rather reflect intrinsic circuit-level changes that precede structural damage.^1,3^ Previous studies have instead reported a reduction in spine density and a shift towards immature spine morphology in the hippocampus of HSV-1-infected mice,^8^ suggesting that early synaptic alterations may differ across brain regions.

Changes in functional connectivity were observed in the absence of marked gliosis or proteinopathy, underscoring their value as early indicators. This functional–molecular dissociation highlights an opportunity for early and more precise identification of individuals at risk of developing Alzheimer's disease.

Our findings suggest that integrating EEG-based connectivity metrics with straightforward motor assessments, such as grip strength or dynamic balance, could offer sensitive and scalable tools for identifying individuals at risk of developing Alzheimer's disease. Consequently, future clinical studies are strongly encouraged to evaluate brain connectivity indices in elderly and MCI patients, allowing for longitudinal tracking and stratification on the basis of grip strength and dynamic balance performance (a test similar to grid walking test) to validate the prognostic value of combined EEG and motor indices.

We preliminary explored this issue by performing parallel studies in humans, which revealed that older participants with MCI showed early signs of motor-functional decline, as indicated by the significantly reduced grip strength when compared to HS. Moreover, MCI generated significantly more strength than Alzheimer's disease, the latter achieving the lowest motor-functional performances, in line with the extant literature.^34,35^ In this context, the finding of MCI patients located between HS and Alzheimer's disease in terms of grip strength provides experimental and translational evidence that such test may serve as a potential marker of early decline that can be employed to spot older adults transitioning towards MCI or more severe cognitive impairment. Importantly, the findings from the motor tests carried out in the animal model prompted and guided the choice of comparable assessments in humans, that is, handgrip strength and 4SST, with the latter being a close proxy of dynamic stability and balance. Such a somewhat ‘translationally inverse’ approach (i.e. from the murine model to humans) allowed us to determine the ability of these tests to classify subjects in a coherent manner between the murine and human models so that clear differences emerged among the three groups (resembling the findings observed in mice), with MCI patients placed between the two, as was the case for mice. While a relatively large ‘corpus’ of literature has validated reduced physical fitness, especially handgrip strength, as a strong predictor of cognitive decline both in humans^36,37^ and in mice, studies have generally focused only on one of the two models. Conversely, the parallel assessment of both mice and humans carried out in this work represents an element of strength, also indicating a commonality between the two models in that motor tests can differentiate healthy from Alzheimer's disease but also from MCI.

It is important to acknowledge certain limitations of the animal study when interpreting and applying our findings from a translational perspective. First, while the murine model used here replicates key aspects of sporadic Alzheimer's disease through recurrent HSV-1 reactivation, it does not fully encompass the complexity of the human condition, including, but not limited to, genetic heterogeneity and environmental factors that are not accounted for in controlled experimental settings. Future research should address sex differences by involving female mice. Finally, the use of other established experimental models of Alzheimer's disease is warranted.

As a last remark, the human component involved a limited number of elderly male participants, which may restrict the generalizability of the findings to the broader population, also preventing powerful subgroup comparisons based on the scores portraying the cognitive function (i.e. Mini-Mental State Examination, Montreal Cognitive Assessment, Addenbrooke's Cognitive Examination-Revised, etc.).

## Conclusions

In summary, we propose that motor deficits, supported by electrophysiological and morphological correlates, constitute robust biomarkers of early Alzheimer's disease–like dysfunction in mice. These results encourage a shift towards integrating motor testing into preclinical screening and support the broader use of functional circuit readouts for early-stage diagnosis and intervention.

## Supplementary Material

fcag128_Supplementary_Data

## Data Availability

The data supporting the findings of this study are available in the article and at DOI https://doi.org/10.5281/zenodo.19912254. Anonymised data may be shared by the senior authors upon reasonable request. Data sharing may be subject to restrictions according to consent and data protection legislation. MATLAB scripts used in the data analysis employed standard functions and packages that are freely available; no new package or function was generated for this study.
