# Supplementary material for: EEG–motor correlation as early Alzheimer’s disease index in herpes simplex virus type-1–infected mice
Source: Brain Commun. 2026 Apr 10;8(3):fcag128. doi: 10.1093/braincomms/fcag128 (PMC13178826; doi:10.1093/braincomms/fcag128)
Supplement: fcag128_Supplementary_Data [file fcag128_supplementary_data.pdf]

Supplementary Materials

Supplementary Materials and methods

Inclusion and Exclusion Criteria

In the NOR test, animals were excluded from the analysis if their total exploration time was less than 20 seconds (n=0 mice). Similarly, in the Y-maze test, animals were excluded if they made fewer than 10 arm entries (n=0 mice). No animals were excluded due to premature death resulting from technical complications.

Grip strength test

The mice were suspended by their tail and allowed to grasp a metal grid connected to a force-measuring instrument (GSM, Bioseb Instrument) with their forepaws, and the maximum strength of the grip was recorded. The values of forelimb strength (g) are reported as the average of three consecutive trials normalized to mouse body weight (g) and were used for statistical analysis.[1,2] Body weight was also recorded and used to evaluate the overall animal welfare.[3]

Grid walking test

The grid walking test is widely used to measure post-stroke motor coordination deficits.[2,4] The apparatus consisted of a 11 mm square openings (320 × 200 × 500 mm) supported by four poles. The mice were allowed to move freely on the grid for 5 min while being recorded with a camera (Panasonic Full HD HC-W580). A mirror placed at a 45° angle under the grid facilitated the simultaneous assessment of the total number of steps and motor coordination errors. A stepping error (foot fault) was defined as any instance in which the forepaws or hindpaws failed to maintain support and slipped through a grid opening. During blinded offline analysis, the number of errors and the total

number of steps performed by each limb were quantified. The percentage of foot faults was calculated as follows: (number of foot faults/total steps) × 100.[2]

NOR

The NOR was used to evaluate the recognition memory. On the first day, the mice were individually subjected to a 10-minute habituation session in an empty arena (45 × 45 cm) to familiarize themselves with the apparatus. On the second day, training was conducted in a single 8-minute session, where two identical objects were placed symmetrically in the arena. On the third day, during the test phase, one of the familiar objects was replaced with a novel object, and the mice were allowed to explore for 8 min.[5] Exploration time (defined as the time the animal's snout was directed at the object from <2 cm) was recorded for the novel and the familiar object. The exploration time recorded during the test phase was then calculated as the preference index, which is the percentage of time spent exploring the novel object compared to the total object exploration (both novel and old objects). To further exclude place preference for one side of the arena, the position of the novel object was alternated on both sides during the test session. The objects and arena were cleaned with a 70% ethanol solution for subsequent tests. All the objects used for the recognition paradigms were of similar sizes. The objects used were bottles and flasks for cell culture filled with clean bedding.

Y-maze test

Spatial working memory was assessed using a Y-maze test. The apparatus consisted of three arms (35 cm long, 5 cm wide, and 15 cm high) arranged at 120° angles to form a Y shape. Each mouse was placed at the distal end of the same designated arm and allowed to explore freely for 8 min, while behavior was recorded using a Panasonic Full HD HC-W580 camera. The arms were labeled as A, B, and C, and the sequence and number of arm entries were recorded. Entry was considered complete when all four paws entered the arm. Spontaneous alternation was defined as sequential entry into three different arms in overlapping triplet sets (e.g., A-B-C, but not A-B-A). The percentage of

spontaneous alternations was calculated as follows: (number of correct alternations) / (total number of entries-2) × 100.[6]

Electrode implantation and LFP recordings

Two weeks before the 2nd reactivation in 2×TS and before the 7th reactivation in 7×TS mice, the animals underwent a surgical procedure for chronic electrode implantation. The mice were anesthetized via an intraperitoneal (i.p.) injection of a mixture of ketamine (87.5 mg/kg) and xylazine (12.5 mg/kg) before being positioned in a stereotaxic frame. A longitudinal scalp incision was used to expose the skull, and six small burr holes were drilled at stereotaxic coordinates corresponding to the FC (+2.5 mm anteroposterior and ± 0.3 lateral from the Bregma), primary motor cortex (+1.4 mm anteroposterior, ± 2 mm lateral from the Bregma) and somatosensory cortex (-0.34 mm anteroposterior, ± 2.4 mm lateral from the Bregma) on both hemispheres. Additionally, two more holes were drilled: one for the placement of the reference electrode (+2.5 mm anteroposterior and -2.5 lateral from the bregma) and the other to facilitate the insertion of a skull screw used as a ground (+2.5 mm anteroposterior and +2.5 lateral from the bregma). Eight stainless-steel filaments were soldered to a multipin socket (NPD-18-DD-GS connector, Omnetics), with six serving as recording electrodes, one serving as the reference, and the last serving as the ground. Each electrode was carefully positioned through the burr holes to ensure electrical contact without damaging the dura mater. This precaution minimized brain trauma and prevented cerebrospinal fluid leakage. The entire implant was then secured with dental light-curing resin (Tetric Evoflow®), providing a minimally invasive approach to reduce potential intracranial pressure fluctuations caused by cranial exposure to atmospheric conditions.[2,4] After surgery, the mice were individually housed, allowed a 7-day recovery period, and closely monitored for any signs of pain or distress. At the end of this period, the animals were individually placed in a recording cage where they were allowed to move freely during the LFP recordings. Each LFP recording session lasted 30 min. Data acquisition was performed using a Cereplex Direct System (Blackrock Microsystems).

LFP data analysis

LFP data were analyzed using MATLAB (MathWorks, Natick, MA, USA) with custom scripts built on the EEGLAB toolbox (Swartz Center for Computational Neurosciences, La Jolla, CA, USA). The signals were bandpass filtered between 0.2 and 47 Hz using a finite impulse response filter. The imported data were segmented into 2-second epochs, and major artifacts, such as those caused by movement or environmental noise, were identified and removed. Following artifact rejection, a minimum of 23 min of usable data per mouse were retained. The LFP data were further analyzed in MATLAB for functional connectivity via EEGLAB-based routines.[4] The functional coupling of LFP rhythms was quantified using Magnitude Squared Coherence (MSCoh), which measures the coherence between all combinations of electrode pairs. The overall coherence across the network was summarized as total MSCoh (TotCoh).[7] The procedure for computing TotCoh included the following steps: (i) for a specified EEG frequency band, the coherence for each electrode was calculated as the mean coherence between that electrode and all others; and (ii) the TotCoh for a given frequency band was then obtained by averaging the coherence values across all electrodes. TotCoh was determined for standard EEG frequency bands: delta (2–4 Hz), theta (4–8 Hz), alpha 1 (8–10.5 Hz), alpha 2 (10.5–13 Hz), beta 1 (13–20 Hz), beta 2 (20–30 Hz), and gamma (30–45 Hz).[2,8]

Immunofluorescence Assays

At the end of the last LFP recording and testing period, the animals were anesthetized via i.p. injection of ketamine (87.5 mg/kg) and xylazine (12.5 mg/kg) and then transcardially perfused with ice-cold phosphate-buffered saline (PBS). Following perfusion, the brain was extracted and postfixed in 4% paraformaldehyde at 4 °C for 48 h and then transferred to a solution of 30% sucrose in 0.1 M PBS. Sagittal brain sections (40 μm thick) were cut using a vibratome (VT1000S, Leica Microsystems, GmbH, Wetzlar, Germany) and then immunoprocessed.

The sections were rehydrated in PBS, blocked at RT for 1 h in a solution containing 1% bovine serum albumin, 10% goat serum, and 0.5% Triton X-100 (Sigma), and incubated overnight with a primary antibody (glial fibrillary acidic protein [GFAP], 1:500; Immunological Sciences mAb #94160; mouse IgG) at 4 °C. The following day, the sections were thoroughly washed and incubated with a secondary antibody (AlexaFluor-488 goat anti-mouse IgG, 1:500).

Immunofluorescence images were acquired using a confocal laser scanning microscope (Nikon A1 MP confocal system, Tokyo, Japan) with identical exposure settings for all the samples. Two 40 µm-thick slices per animal were used for the analysis. Images (1024 × 1024 pixels) were acquired at 20× magnification with a Nikon A1 MP confocal system (Tokyo, Japan). GFAP expression was quantified to assess astrogliosis. Images were processed and analyzed with ImageJ software: fluorescence intensity was calculated by applying an automatic Otsu threshold to isolate GFAP-positive areas, followed by measurement of integrated density. The fluorescence intensity was measured in the FC.

Western blotting

The mice were deeply anesthetized and transcardially perfused with ice-cold PBS. The FC was rapidly dissected on ice and snap-frozen in liquid nitrogen. Total proteins were extracted via ice-cold RIPA buffer prepared in-house (50 mM Tris-HCl pH 7.4, 150 mM NaCl, 1% Triton X-100, 0.5% sodium deoxycholate, 0.1% SDS) freshly supplemented with protease and phosphatase inhibitors. The lysates were centrifuged at 14,000×g for 15 minutes at 4 °C; the supernatants were collected and stored at −80 °C until use.

Protein concentrations were determined with a micro BCA protein assay kit (#ZB382871, Thermo Fisher Scientific). Equal amounts of protein (30 µg) were loaded onto 12% Tris-glycine polyacrylamide gels (Bio-Rad) for electrophoretic separation. Precision Plus Protein™ Dual Color Standards (Bio-Rad) were used as molecular mass markers. Proteins were transferred onto polyvinylidene fluoride membranes (Millipore) and stained with Ponceau S solution to verify uniform protein loading.

The membranes were blocked with 5% nonfat dry milk in Tris-buffered saline with 0.1% Tween-20 (TBST) for 1 hour at RT and then incubated overnight at 4 °C with the following primary antibodies: anti-Tau (1:1000, #sc-21796 Santa Cruz Biotechnology), anti-phospho-Tau (Ser199) (1:1000, #29957 Cell Signaling Technology), and anti-glyceraldehyde-3-phosphate dehydrogenase (GAPDH) (1:2000, #AB9484 Abcam). After three 10-minute washes in TBST, the membranes were incubated for 1 hour at RT with HRP-conjugated secondary antibodies (Cell Signaling Technology #7074S, 1:2000). The membranes were washed again, and the immunoreactive bands were visualized with enhanced chemiluminescence (ECL; Thermo Fisher Scientific) and imaged using a ChemiDoc Imaging System (Bio-Rad). Band intensities were quantified by densitometry using UVITECH software and normalized to that of GAPDH, which was used as a loading control. Each sample was analyzed in technical triplicate to ensure measurement reliability.

Golgi-Cox staining and dendritic spine density analysis

The brains were processed for Golgi-Cox staining using the FD Rapid GolgiStain™ Kit (FD NeuroTechnologies, Columbia, MD, USA) following the manufacturer's instructions. Briefly, the brains were immersed in impregnation solutions, and after 16 days, they were rapidly frozen in liquid nitrogen and stored overnight at −80 °C. Sagittal sections (100 μm) were obtained using a cryostat (Slee MEV Cryostat; Slee Medical GmbH, Germany) at −20 °C. The sections were mounted on gelatin-coated slides, stained, dehydrated, cleared in xylene, and coverslipped with Eukitt® mounting medium.

Pyramidal neurons from layers II/III of the motor cortex were selected on the basis of the following inclusion criteria: complete and uniform impregnation, absence of background precipitate, planar orientation of dendritic arborization, and relative isolation from neighboring stained cells. Dendritic spine density was analyzed on the second- and third-order branches of both the apical and basal dendrites. The primary shafts and the initial segment (first few micrometers) of each branch were excluded from the analysis because of the typically low spine density in the proximal regions.

Quantification of ~20 µm-long dendritic segments was performed by using Neurolucida 7.5 software (MicroBrightField, Williston, VT, USA) connected to a motorized-stage Zeiss microscope equipped with a high-resolution digital camera. The spine density was calculated by using Neurolucida Explorer as the number of spines/µm length of the dendritic segment. Thirty neurons per group (n=3 mice/group) were analyzed, with 10 neurons randomly selected per animal.

Supplementary Figures

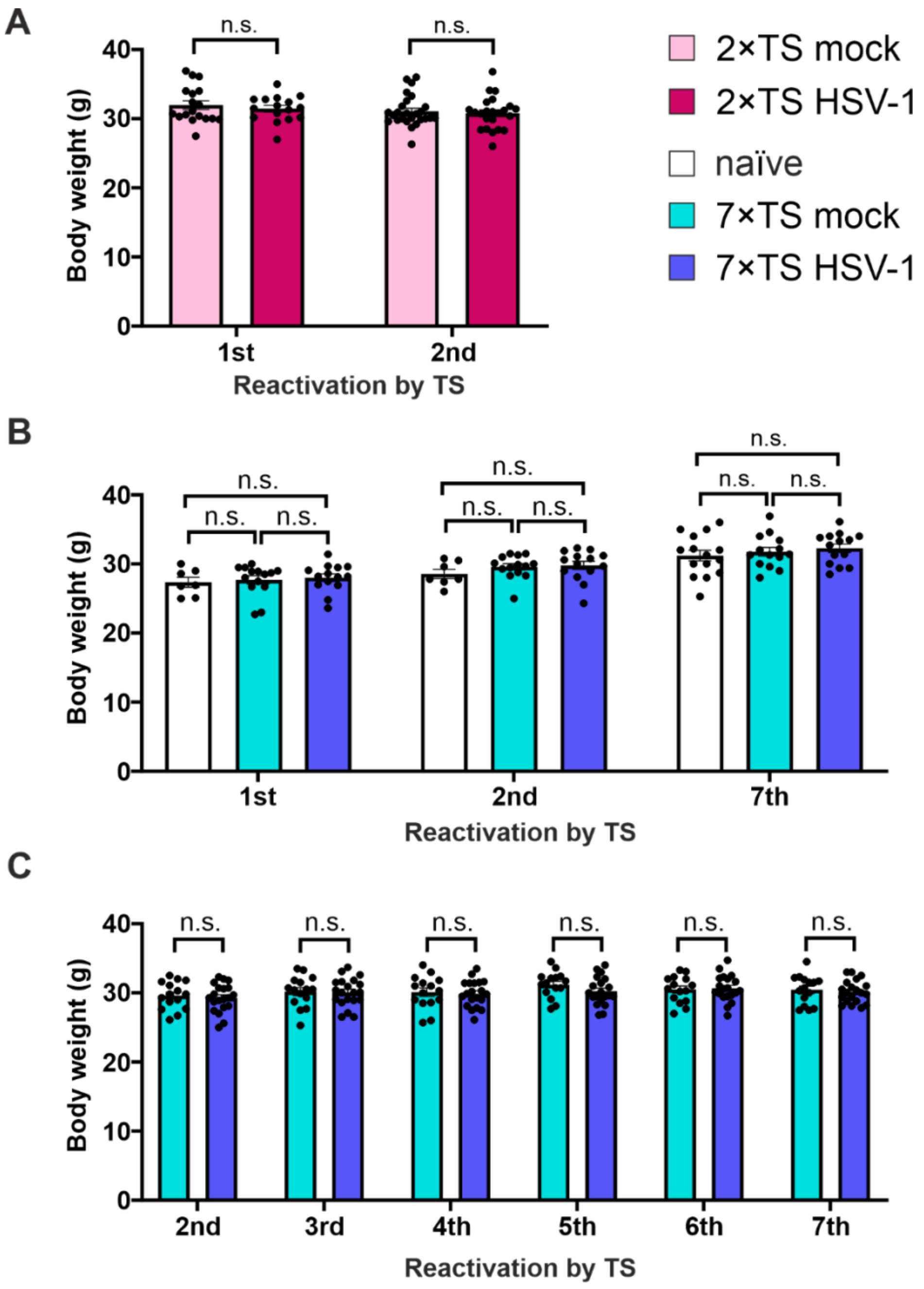

Supplementary Figure 1. Evaluation of the mouse body weight after each TS in the different experimental groups. (**A**) Body weight of 2×TS mock and 2×TS HSV-1 mice after the 1st (2×TS mock: n=18; 2×TS HSV-1: n=16) and 2nd TS (2×TS mock: n=25; 2×TS HSV-1: n=23). (**B**) Body weight of naïve, 7×TS mock and 7×TS HSV-1 mice after 1st, 2nd (naïve: n=7; 7×TS mock: n=14; 7×TS HSV-1: n=14) and 7th TS (naïve: n=15; 7×TS mock: n=14; 7×TS HSV-1: n=14). (**C**) Body

weight of 7×TS mice that underwent grip strength test after the 2nd, 3rd, 4th, 5th, 6th and 7th TS (7×TS mock: n=15; 7×TS HSV-1: n=20). Data are presented as mean ± SEM. Two-way RM ANOVA (repetition factor: number of TS), Bonferroni post hoc; n.s., not significant. Each dot represents an individual animal.

Regarding the 2×TS groups, statistical analysis revealed no effect of group ($F_{(1, 47)}$= 0.5375; P=0.4671) and no effect of the number of TS ($F_{(1, 32)}$=1.897; P=0.1779; panel A). In the 7×TS groups, statistical analysis revealed no effect of group (first cohort: $F_{(2, 40)}$=0.1862; P=0.8308; second cohort: $F_{(1, 33)}$=0.2213; P=0.6411), but there was a main effect of the number of TS (first cohort: $F_{(2, 64)}$=63.31; P<0.0001; second cohort: $F_{(5, 165)}$=7.891; P<0.0001; panels 1B,C) related to the physiological increase in body weight over time.

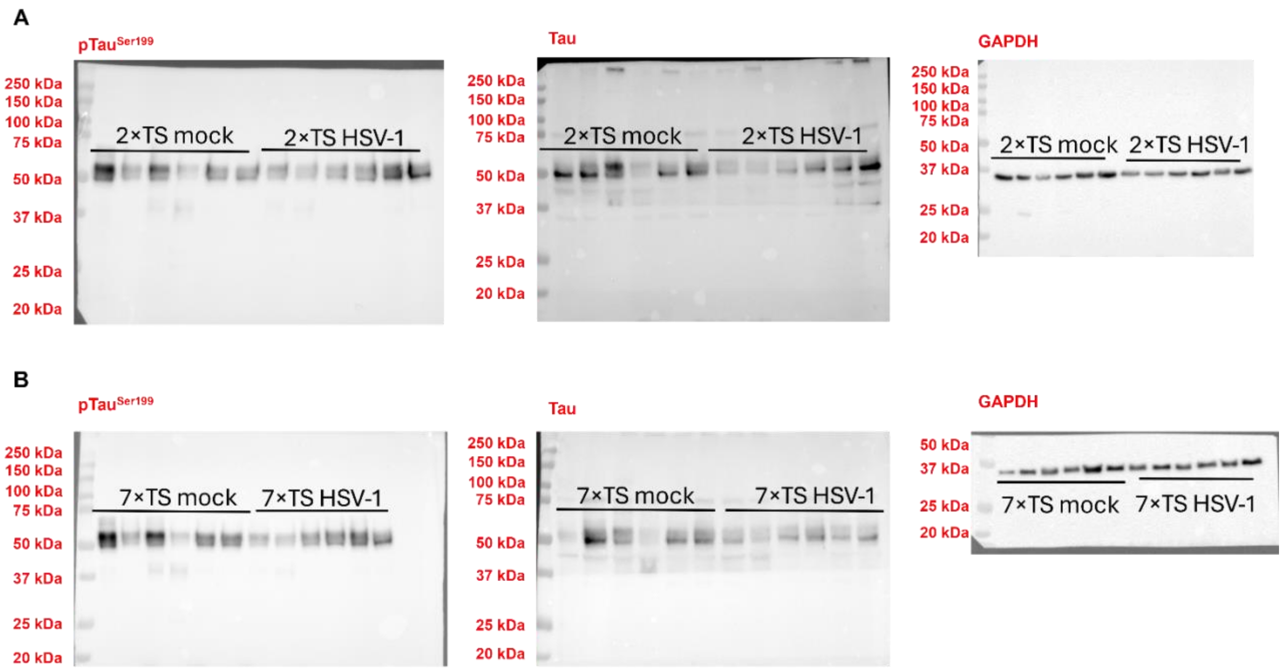

Supplementary Figure 2. Uncropped Western blots corresponding to data presented in Figure 4 of the main manuscript. Uncropped Western blots corresponding to data presented in Figure 4C (**A**) and in Figure 4D (**B**).

References

1. Barbati SA, Cocco S, Longo V, et al. Enhancing plasticity mechanisms in the mouse motor cortex by anodal transcranial direct-current stimulation: the contribution of nitric oxide signaling. *Cereb Cortex.* 2020;30(5):2972–2985.

2. Longo V, Barbati SA, Re A, et al. Transcranial direct current stimulation enhances neuroplasticity and accelerates motor recovery in a stroke mouse model. *Stroke*. 2022;53(5):1746–1758.

3. Talbot SR, Biernot S, Bleich A, et al. Defining body-weight reduction as a humane endpoint: a critical appraisal. *Lab Anim.* 2020;54(1):99–110.

4. Miraglia F, Pappalettera C, Barbati SA, et al. Brain complexity in stroke recovery after bihemispheric transcranial direct current stimulation in mice. *Brain Commun.* 2024;6(3):fcae137.

5. Cocco S, Rinaudo M, Fusco S, et al. Plasma BDNF levels following transcranial direct current stimulation allow prediction of synaptic plasticity and memory deficits in 3×Tg-AD mice. *Front Cell Dev Biol.* 2020;8:541.

6. Kraeuter AK, Guest PC, Sarnyai Z. The Y-maze for assessment of spatial working and reference memory in mice. *Methods Mol Biol.* 2019;1916:105–111.

7. Vecchio F, Pappalettera C, Miraglia F, et al. Prognostic role of hemispherical functional connectivity in stroke: a study via graph theory versus coherence of electroencephalography rhythms. *Stroke.* 2023;54(2):499–508.

8. Cacciotti A, Pappalettera C, Miraglia F, Rossini PM, Vecchio F. EEG entropy insights in the context of physiological aging and Alzheimer's and Parkinson's diseases: a comprehensive review. *Geroscience.* 2024;46(6):5537–5557.
